# Small RNA Bidirectional Crosstalk During the Interaction Between Wheat and *Zymoseptoria tritici*

**DOI:** 10.3389/fpls.2019.01669

**Published:** 2020-01-08

**Authors:** Xin Ma, Jasmin Wiedmer, Javier Palma-Guerrero

**Affiliations:** Plant Pathology, Institute of Integrative Biology, ETH Zurich, Zurich, Switzerland

**Keywords:** *Triticum aestivum* L, *Zymoseptoria tritici*, small RNAs, RNAi, sRNA-seq, degradome analysis

## Abstract

Cross-kingdom RNA interference (RNAi) has been shown to play important roles during plant–pathogen interactions, and both plants and pathogens can use small RNAs (sRNAs) to silence genes in each other. This bidirectional cross-kingdom RNAi was still unexplored in the wheat–*Zymoseptoria tritici* pathosystem. Here, we performed a detailed analysis of the sRNA bidirectional crosstalk between wheat and *Z. tritici*. Using a combination of small RNA sequencing (sRNA-seq) and microRNA sequencing (mRNA-seq), we were able to identify known and novel sRNAs and study their expression and their action on putative targets in both wheat and *Z. tritici*. We predicted the target genes of all the sRNAs in either wheat or *Z. tritici* transcriptome and used degradome analysis to validate the cleavage of these gene transcripts. We could not find any clear evidence of a cross-kingdom RNAi acting by mRNA cleavage in this pathosystem. We also found that the fungal sRNA enrichment was lower *in planta* than during *in vitro* growth, probably due to the lower expression of the only Dicer gene of the fungus during plant infection. Our results support the recent finding that *Z. tritici* sRNAs cannot play important roles during wheat infection. However, we also found that the fungal infection induced wheat sRNAs regulating the expression of specific wheat genes, including auxin-related genes, as an immune response. These results indicate a role of sRNAs in the regulation of wheat defenses during *Z. tritici* infection. Our findings contribute to improve our understanding of the interactions between wheat and *Z. tritici*.

## Introduction

Small RNAs (sRNAs) are short non-coding RNAs (approximately 20–30 nucleotides in length) that play important roles in the regulation of gene expression and genome stability in eukaryotic organisms (Moazed, 2009). The three major classes of sRNAs are microRNAs (miRNAs), small interfering RNAs (siRNAs), and PIWI-associated RNAs (piRNAs) (Carthew and Sontheimer, 2009; Moazed, 2009). While many miRNAs and siRNAs are conserved among plants, piRNAs have so far only been found in animal cells and are necessary in the development of germ cells by silencing transposons or other genetic elements (Vagin et al., 2006; Siomi et al., 2011; Axtell, 2013).

In plants, miRNAs (~21 nt) are produced from imperfect fold-back structures of self-complementary non-coding transcripts, originated from endogenous loci, and they regulate target mRNAs by posttranscriptional gene silencing (PTGS). In contrast, siRNAs derive from double-strand RNA (dsRNA) with near-perfect complementarity, and they can be both of *endogenous* origin, e.g., inverted repeat (IR) and dsRNA synthesized by endogenous RNA-dependent RNA polymerases (RDRs) (Allen et al., 2005; Felippes and Weigel, 2009; Fei et al., 2013; Creasey et al., 2014), or *exogenous* origin, e.g., viral intermolecular replication intermediates or intramolecular secondary structures. siRNAs guide either PTGS (mediated by 21/22-nt siRNAs) or RNA-directed DNA methylation (RdDM) often resulting in transcriptional gene silencing (TGS; mediated by 24-nt siRNAs) (Matzke and Mosher, 2014; Borges and Martienssen, 2015). While each sRNA pathway possesses specific characteristics in terms of biogenesis and accessory proteins, the basic mechanism of RNA silencing shares few consensus biochemical steps: i) initiation by dsRNA, ii) dsRNA processing to generate the mature sRNAs by Dicer (or Dicer-like, DCL) proteins, and iii) sRNA incorporation into Argonaute (AGO) proteins to interact with target mRNA or DNA in order to execute their silencing functions (Liu et al., 2004; Hutvagner and Simard, 2008; Bologna and Voinnet, 2014).

sRNAs are involved in plant development, reproduction, and genome reprogramming and contribute to the phenotypic plasticity of plants (Borges and Martienssen, 2015). Besides, plant sRNAs also mediate the response to abiotic stresses, such as water stress, sulfate stress, phosphate starvation, and cold stress (Sunkar and Zhu, 2004; Sunkar et al., 2007; Park et al., 2010; Guleria et al., 2011; Kamthan et al., 2015). Plants as well employ RNA interference (RNAi) as defense strategies against biotic stress (Ruiz-Ferrer and Voinnet, 2009; Zhang et al., 2011; Weiberg et al., 2014). In *Arabidopsis*, miR393 is induced during bacterial infection, and it suppresses the auxin signaling by targeting auxin response TIR1 and three related F-box genes to inhibit pathogen growth (Achard et al., 2007; Chen et al., 2007; Navarro et al., 2008; Grant and Jones, 2009). Besides, plant miRNAs can also regulate NBS-LRR transcripts as an important immune response to pathogens. (Zhai et al., 2011; Li et al., 2012; Shivaprasad et al., 2012; Fei et al., 2013).

In fungi, RNAi is an ancient host defense mechanism against viruses and transposons invasion (Chang et al., 2012; Nicolas and Ruiz-Vazquez, 2013; Torres-Martinez and Ruiz-Vazquez, 2017). However, no miRNAs, but only miRNA-like RNAs (milRNAs), have been identified in fungi (Lee et al., 2010; Goodwin et al., 2011), which share similarities with conventional miRNAs, but are synthesized by different mechanisms (Lee et al., 2010). The first RNAi process in fungi, called quelling, was found in *Neurospora crassa*. Quelling is triggered by repetitive transgenes during mitotic growth, and it is essential for suppressing transposons replication (Romano and Macino, 1992; Nolan et al., 2005). QiRNAs, a class of Quelling-Deficient 2 (QDE2) interacting sRNAs, are specifically induced when *Neurospora* is treated with a DNA-damaging agent (Lee et al., 2009). Additionally, sRNAs are also required for fungal growth and pathogenesis in *Magnaporthe oryzae* (Raman et al., 2017).

RNAi has recently been reported to play important roles in plant and pathogen interactions. In host-induced gene silencing (HIGS), accumulation of double-stranded or antisense RNAs in barley and wheat can target and silence genes in the fungal pathogen *Blumeria graminis* as an effective defense strategy (Nowara et al., 2010). This cross-kingdom RNAi has also been shown to happen in a natural way. A fungal pathogen, *Botrytis cinerea*, can hijack host plant defenses by using small RNAs that bind to the plant AGO1 and silence plant defense-related genes (Weiberg et al., 2013). A novel milRNA has been identified in *Puccinia striiformis* f. sp. *tritici* (Pst) that silences a wheat pathogenesis-related 2 gene and influences the plant immunity (Wang et al., 2017). Plants can also use the same RNAi mechanisms to defend against pathogenic fungi, e.g., cotton plants can export their miRNAs into the fungal pathogen *Verticillium dahliae* and inhibit the expression of virulence genes during the infection cycle (Wang et al., 2016; Zhang et al., 2016). In addition, a recent study has shown that *Arabidopsis* can send sRNAs in extracellular vesicles to fungal pathogens, revealing how sRNAs travel from hosts to fungal pathogens (Cai et al., 2018).

*Zymoseptoria tritici* causes septoria tritici blotch, which is an important disease of wheat worldwide and the most damaging disease on wheat in Europe (Hardwick et al., 2001; O’Driscoll et al., 2014). After penetrating wheat leaves through stomata, *Z. tritici* starts the complex infection cycle characterized by a long period of asymptomatic growth known as a latent period that can last up to 2 weeks (Sanchez-Vallet et al., 2015). This latent period is followed by a rapid switch to necrotrophic growth in which a large number of plant cells are killed in a 2- to 3-day period, and it is accompanied by a rapid accumulation of fungal biomass inside the plants (Rudd et al., 2015). Even though there are many predicated candidate effectors in *Z. tritici* (Mirzadi Gohari et al., 2015; Rudd et al., 2015; Palma-Guerrero et al., 2016), so far, only LysM has been confirmed to be required for virulence, and it can suppress PAMP-triggered immunity during the infection (Marshall et al., 2011; Mirzadi Gohari et al., 2015). Therefore, the mechanisms underlying *Z. tritici* pathogenicity still remain largely unknown. In previous studies, we have analyzed the transcriptomes of both wheat and *Z. tritici* during the infection (Palma-Guerrero et al., 2017; Ma et al., 2018b). These studies revealed the mutual transcriptomic responses between wheat and *Z. tritici* during the interactions, which, together with other transcriptomics studies in this pathogen, shows that a whole genome-wide reprogramming of gene expressions happens in both plant and fungus (Rudd et al., 2015; Palma-Guerrero et al., 2017; Ma et al., 2018b). A recent study reported that the deletion of RNAi-related genes in *Z. tritici* is dispensable for full infection of wheat. Conversely, they also showed that HIGS is not effective to control *Z. tritici* (Kettles et al., 2019). This work indicates that *Z. tritici* sRNAs do not play important roles in a cross-kingdom RNAi manner, contrary to previous studies in other fungal plant pathogens (Weiberg et al., 2013; Wang et al., 2017). But how wheat sRNAs take part in this interaction still remains unexplored. The investigation of this natural bidirectional RNAi between wheat and *Z. tritici* at the same time in the same samples can provide a detailed scenario of this hidden interactions.

Here, we analyzed the small RNAs produced during wheat infection by *Z. tritici* during the latent period and the transition phase of the disease cycle to identify both the *Z. tritici* sRNAs and the wheat sRNAs and their degraded targets. We found fungal sRNAs induced during infection and predicted their targeted genes in the wheat transcriptome. But cleavage of these predicted targets could not be confirmed by degradome analysis. Additionally, the expression of the fungal Dicer gene was downregulated and barely expressed *in planta*. Conversely, wheat sRNAs involved in the regulation of auxin signaling were also induced in response to *Z. tritici*. Thus, our results suggest that the cross-kingdom RNAi in wheat and *Z. tritici* pathosystem does not lead to detectable mRNA degradation by PTGS during infection. These findings support the recent published work of fungal sRNAs in wheat and *Z. tritici* pathosystem (Kettles et al., 2019). However, we also show that wheat sRNAs can be used as an immune response to the infection by *Z. tritici*.

## Materials and Methods

### Samples Collection and RNA Extractions

The total RNAs from leaf samples of wheat cultivar Drifter infected with the *Z. tritici* 3D7 strain at 7, 12, and 14 days post-infection (dpi) were obtained in a previous study from our group (Palma-Guerrero et al., 2017). The RNAs from uninfected mock samples at 7, 12, and 14 dpi that serve as controls were obtained in this study by using the control samples generated in the same previous study (Palma-Guerrero et al., 2017). The RNA samples of the fungus growing *in vitro* were generated in a different previous study (Francisco et al., 2018). Three replicates per sample with the highest RNA quality according to Bioanalyzer 2100 (Agilent) were used as biological replicates for all the library preparations and sequencing. All the raw sequencing data were deposited into the NCBI Short Read Archive under the accession number SRP154808.

### Small-RNA-Seq Analysis

The small-RNA-seq libraries were constructed using the TruSeq small-RNA kit and sequenced by HiSeq4000 by the Functional Genomic Center of Zurich (FGCZ). Reads were trimmed to remove sequencing adapters and keep only insert with length ≥15 using Trimmomatic-0.36 (Bolger et al., 2014) with the settings “ILLUMINACLIP:smallRNA-SE.fa:2:30:10 LEADING:2 TRAILING:2 SLIDINGWINDOW:4:15 MINLEN:15”. The adapter sequence in smallRNA-SE.fa is “TGGAATTCTCGGGTGCCAAGG”.

### Wheat sRNA Predictions

All the clean reads were collapsed using fastx-ToolKit (Gordon and Hannon, 2010). Wheat transfer RNAs (tRNAs), ribosomal RNAs (rRNAs), and small nucleolar RNAs (snoRNAs) were removed. The sequences of tRNAs, rRNAs, and snoRNAs were extracted from Triticum_aestivum.TGACv1.ncrna.fa on Ensembl plant (Kersey et al., 2018).

We used miRDeep-p (Yang and Li, 2011) and ShortStack (Shahid and Axtell, 2014; Johnson et al., 2016) for the wheat miRNA predictions. For miRDeep-p, all the filtered reads were mapped against wheat genome International Wheat Genome Sequencing Consortium (IWGSC) RefSeq v1.0 (Appels et al., 2018) using bowtie (Langmead et al., 2009), allowing no mismatches. The mapped reads from all the samples including infected samples and mocks were merged together and collapsed. We used the mapped reads to retrieve miRNAs annotated in IWGSC RefSeq v1.0 annotations (Appels et al., 2018). We used miRDeep-P (Yang and Li, 2011) to predict wheat miRNAs, setting the length of candidate precursors as 300 nt. A customized Perl script was used to filter the miRNAs based on the new criteria of plant miRNA annotation (Axtell and Meyers, 2018). Basically, 1) both the miRNA and miRNA* are predicted and expressed in the sequencing data; 2) the expression of miRNA is more than 10 reads per million in at least one of the samples; 3) the length of mature miRNAs should be between 20 and 24 nt; 4) the mismatches between miRNA and miRNA* are under 5; 5) the asymmetric mismatches between miRNA and miRNA* are under 3; and 6) the length of precursor should be under 300 nt.

We as well used ShortStack (Shahid and Axtell, 2014; Johnson et al., 2016) to predict the wheat sRNA loci with the settings “—mismatches 0 –mincov 0.5rpm.” Three replicates of each condition were merged together for the ShortStack alignment and annotation. Only the sRNA candidate loci with expressions of at least 10 rpm in at least two replicates of each condition were kept. Then the major siRNAs and miRNAs from every condition were summarized together. We named every unique siRNA and miRNA reads as miRNA-uniq or siRNA, and with ascending order numbers. We combined the annotations of miRNAs from *Arabidopsis*, rice, and wheat from miRBase and the annotations of wheat miRNA from IWGSC together to make a customized database of the known plant miRNAs (Griffiths-Jones, 2004; Griffiths-Jones et al., 2006; Griffiths-Jones et al., 2008; Kozomara and Griffiths-Jones, 2011; Kozomara and Griffiths-Jones, 2014). All the unique mature sequences of predicted miRNAs were used to blast (*e*-value under 10) against the customized database to detect if the miRNAs were previously annotated (Camacho et al., 2009).

All the sequences of mature sRNAs were used to count sRNA expression in each sample. We used TMM (trimmed mean of *M* values) (Robinson and Oshlack, 2010) to normalize the library sizes and RPM (reads per million) to describe the sRNA expression. The differentially expressed sRNAs were called by edgeR (Robinson et al., 2010) using log(fold change) ≥1 (or −1 or less) and FDR < 0.05.

### Fungal sRNA Predictions

The fungal tRNAs, rRNAs, and snoRNAs were removed from clean reads. The annotations of tRNAs, rRNAs, and snoRNAs were extracted from Ensembl fungi (Goodwin et al., 2011; Kersey et al., 2018). The published genome reference of 3D7 was used in the analysis (Plissonneau et al., 2016). Then the filtered reads were used to predict fungal sRNAs with ShortStack using the same strategy as described above for the plant. The expression of the fungal sRNAs were obtained as described above for the plant sRNAs.

### SRNA Target Predictions and Degradome Analysis

The wheat and *Z. tritici* sRNAs upregulated or highly expressed (RPM ≥ 100) at 7 or 12 dpi were used to predict the target genes in wheat and *Z. tritici* transcriptome using psRNATarget (Dai et al., 2018). Default parameters from Schema V2 (2017 release) were used, but the expectation value was set at 4.5. Only the targeted genes that were significantly downregulated while the sRNAs showed upregulation or were constantly highly expressed were considered as the predicted target genes.

To confirm the cleavage of the predicted target genes, we generated modified PARE libraries with RNAs from the fungus (*in vitro*), infected wheat leaves (7 and 12 dpi), and mock leaves at the same time points. The samples for the modified PARE libraries were the same ones used for the small RNA sequencing (sRNA-seq) libraries. Poly(A)+ RNA was isolated from the RNA samples. Illumina TruSeq sequencing adapter was ligated to the 5′P ends of the degraded mRNA. Next, first-strand complementary DNA (cDNA) synthesis was performed using a N6 randomized primer. After RNA hydrolysis, the 3′ Illumina TruSeq sequencing adapter was ligated to the 5′ ends of the antisense first-strand cDNA fragments. The 5′ cDNA fragments were finally amplified with PCR using the Illumina TruSeq sequencing adapters as primers. The libraries were sequenced on an Illumina Next Seq 500 single end at 1 × 75 bp using a Truseq SBS kit v3-HS (Illumina, Inc. San Diego, CA, USA, NCBI-SRA project number SRP154808).

The raw sequencing data were trimmed to remove adapters and trimmed length ≥15 using Trimmomatic-0.36 (Bolger et al., 2014) with the settings “ILLUMINACLIP : TruSeq3-SE.fa:2:30:10 LEADING:10 TRAILING:10 SLIDINGWINDOW:4:15 MINLEN:15”. The clean reads were analyzed using CleaveLand4 (Brousse et al., 2014) with the parameters “-c 3 -p 0.05 -r 0.7” for every possible condition. Only the results with a *P* value under 0.05 and a minimum free energy (MFE) ratio higher than 0.7 were regarded as significant results. We only considered the degradome peaks in categories 0, 1, 2, and 3. These categories are described in Cleaveland 4.

### mRNA-Seq Sequencing and Analysis

The microRNA sequencing (mRNA-seq) data from the wheat samples infected with *Z. tritici* were generated in a previous study (Palma-Guerrero et al., 2017), and they are available at the National Center for Biotechnology Information (NCBI) Short Read Archive (SRA) under the project SRP077418. The new mRNA-seq data from the 3D7 isolate grown *in vitro* and plant mocks at 7 and 12 dpi were generated in this study using Illumina HiSeq2500 at 2 × 125 bp (NCBI-SRA project number SRP154808). All the raw sequencing reads were trimmed using Trimmomatic-0.36 as described before. The clean reads were aligned against wheat genome and *Z. tritici* genome separately using tophat v2.1.1 (Kim et al., 2013). HTSeq-count v0.6.1 was used to calculate the gene counts (Anders et al., 2015). We used TMM implemented in Bioconductor EdgeR v3.12.0 to normalize the library sizes and gene expressions (Robinson et al., 2010; Robinson and Oshlack, 2010). Differentially expressed genes were defined using EdgeR. The Benjamin–Hochberg false discovery rate correction was used to adjust *P* values based on the exact Fisher test (Robinson et al., 2010). The genes with a log fold change ≥1 (or −1 or less) and adjusted *P* value <0.05 were determined as the differentially expressed genes. The Gene Ontology (GO) analysis was performed with TopGO v2.32.0 (Alexa and Rahnenfuhrer, 2016).

### Northern Blot Analysis

Total RNA from wheat leaves infected by *Z. tritici* at 12 dpi and uninfected wheat leaves at the same time point were used for Northern blot. Of total RNA, 7.5 µg was separated on 17.5% polyacrylamide–urea gels, electro-transferred to a Hybond-NX membrane (GE Healthcare), and cross-linked *via* 1-ethyl-3-(3-dimethylaminopropyl) carbodiimide-mediated chemical cross-linking, as previously described (Bologna et al., 2018). Oligonucleotides were end-labeled by incubation with T4 PNK (Thermo Scientific) in the presence of [γ-32P] dATP. Multiple small RNAs were hybridized on individual membranes by stripping twice with boiling 0.1% SDS and re-probing. The probe used for miRNA-uniq-1, miRNA-uniq-113, and miRNA-uniq-133 were: “GGGGAATGAAGCCTGGTCCGA,” “GTGCTCACTCTCTTCTGTCA,” and “TGGCATACAGGGAGCCAGGCA,” respectively. U6 was used as positive control.

### Quantification of Relative Gene Expression by Quantitative Real-Time RT-qPCR

The transcription levels of four candidate genes predicted to be cleaved by sRNAs were analyzed using quantitative reverse transcription polymerase chain reaction (qRT-PCR). Six hundred nanograms of total RNA from leaves infected by *Z. tritici* at 12 dpi and uninfected mock leaves was retrotranscribed by using the Revert First Strand cDNA Synthesis kit (Thermo Fisher) following the manufacturer’s instructions. Primer efficiencies were determined by qPCR amplification of a serial dilution of genomic DNA. A melting analysis was performed at the end of the amplification to confirm single product amplification, and non-retrotranscribed RNA was used to discard DNA contamination in the RNA samples. For each candidate gene, three biological and three technical replications were used. Relative expression was calculated from the Ct values of the genes during infection compared to the mock samples using wheat 18s rRNA gene for normalization.

## Results

### *Z. tritici* Produces sRNAs During Wheat Infection

To explore the roles of RNAi in the wheat–*Z. tritici* pathosystem, we analyzed sRNA-seq libraries from samples of wheat leaves infected by *Z. tritici* after 7, 12, and 14 dpi. The corresponding wheat mock leaves at the same points and *Z. tritici* grown *in vitro* were used as controls. Three biological replicates were used for each condition. According to the description of the disease cycle (Rudd et al., 2015; Sanchez-Vallet et al., 2015), our dataset covered the latent period (7 dpi), transition period (12 dpi), and the early necrotrophic period (14 dpi) of the 3D7 strain disease cycle (Palma-Guerrero et al., 2017).

Reads with similarity to tRNAs, rRNAs, and snoRNAs of wheat and *Z. tritici* were filtered and the remaining reads were aligned against the wheat and the *Z. tritici* genome separately to distinguish the plant and fungal sRNA reads (**Table 1**). In total, we obtained 0.3–1.1 million fungal sRNA reads in the infected samples and 1.8–2.8 million fungal sRNA reads in the *in vitro* samples. Between 3.4 and 18.1 million plant sRNA reads were obtained in the infected samples and 1.9–6.5 million plant sRNA reads in the mock samples. The number of fungal sRNA reads increased during the infection process while the number of plant sRNA reads decreased, which is consistent with the disease progression on the infected leaves.

**Table 1:** sRNA seq data summary.

Conditions	Library ID	Total clean reads	Fungal sRNA reads	Fungal sRNA ratios (%)	Plant sRNA reads	Plant sRNA ratios (%)
**Infection samples**	3D7-7dpi-2	71,764,255	339,262	0.47	18,186,405	25.34
	3D7-7dpi-8	75,365,538	385,127	0.51	17,763,333	23.57
	3D7-7dpi-10	48,440,536	306,834	0.63	9,039,173	18.66
	3D7-12dpi-2	65,969,573	460,119	0.70	12,378,083	18.76
	3D7-12dpi-5	65,138,243	653,052	1.00	10,109,163	15.52
	3D7-12dpi-6	45,790,107	317,915	0.69	10,725,249	23.42
	3D7-14dpi-4	31,090,290	901,839	2.90	3,783,930	12.17
	3D7-14dpi-5	34,372,386	577,053	1.68	5,365,083	15.61
	3D7-14dpi-10	30,549,120	1,108,689	3.63	3,406,298	11.15
**Fungal *in vitro***	3D7-vitro-1	16,547,092	2,865,958	17.32	–	–
	3D7-vitro-2	12,318,911	2,191,788	17.79	–	–
	3D7-vitro-3	9,515,263	1,819,328	19.12	–	–
**Plant mocks**	mock-7dpi-C1	17,166,095	–	–	4,586,381	26.72
	mock-7dpi-C2	21,565,083	–	–	5,321,432	24.68
	mock-7dpi-C3	24,379,228	–	–	6,568,124	26.94
	mock-12dpi-C1	20,048,211	–	–	3,884,980	19.38
	mock-12dpi-C2	23,466,121	–	–	4,787,936	20.40
	mock-12dpi-C4	14,114,494	–	–	3,065,456	21.72
	mock-14dpi-C1	8,993,244	–	–	1,902,557	21.16
	mock-14dpi-C2	28,168,941	–	–	6,222,211	22.09
	mock-14dpi-C4	11,895,554	–	–	2,884,932	24.25

sRNA ratios represent the percentage of fungal or plant sRNAs among the total amount of clean reads.

**Figure 1:**
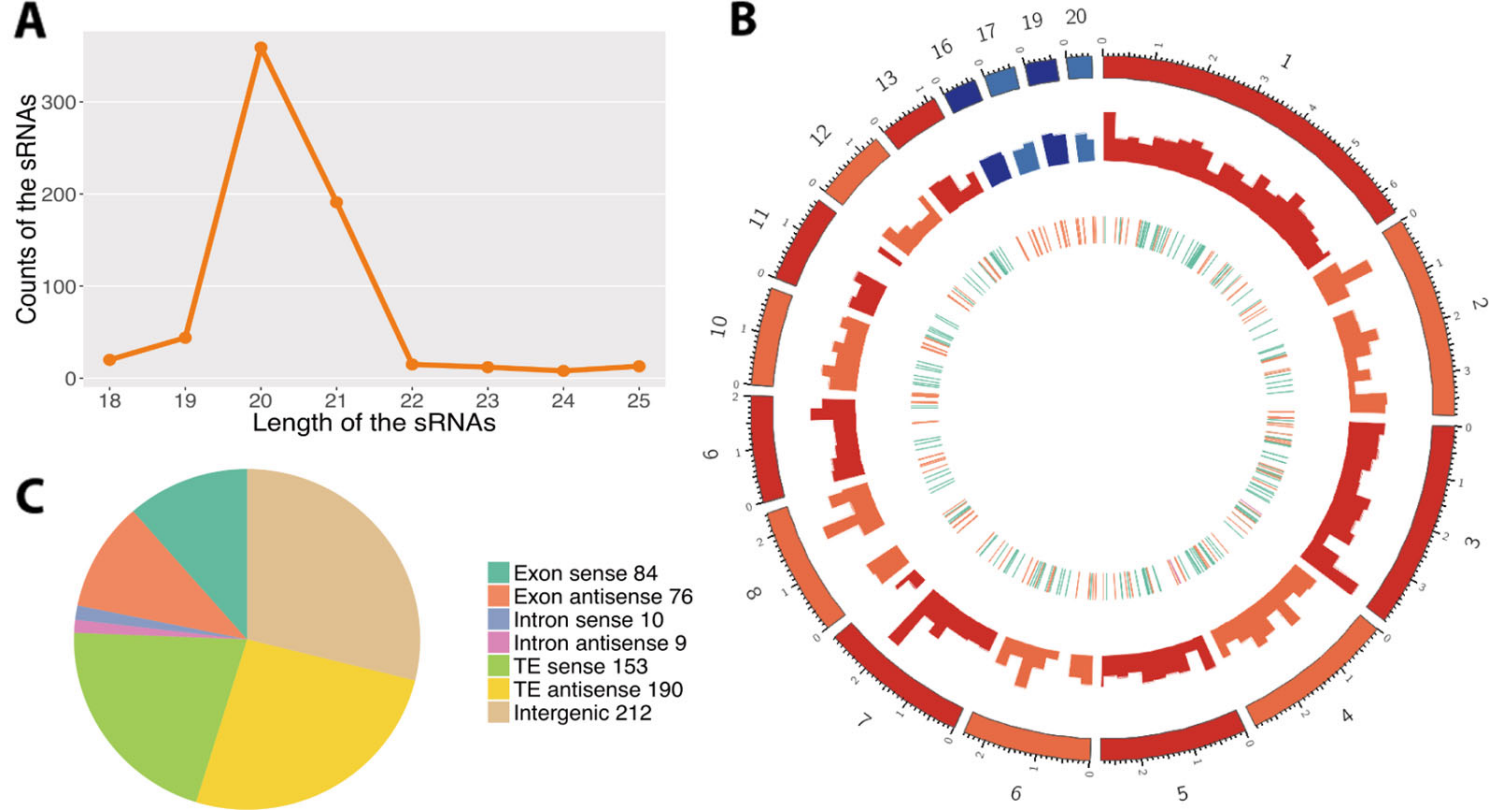
Predictions of the fungal small RNAs (sRNAs). **(A)** Length distribution of the predicted fungal sRNAs. The *x*-axis indicates the lengths of the sRNAs and the *y*-axis indicates the counts of the sRNAs. **(B)** Distribution of the fungal sRNAs across the fungal genome. The *outer layer* shows the chromosomes of the fungal genome. The *red colors* are the core chromosomes and the *blue colors* are the accessory chromosomes. Accumulation of fungal sRNAs (log2-transformed) is shown in the *middle layer*. The *inner layer* shows the genomic elements where fungal sRNAs come from. The *green ones* reveal the sRNAs come from the gene region, the *orange ones* from transposable element (TE), and the *pink ones* from the overlap of gene and TE regions. **(C)** Origin of the fungal sRNAs. The *different colors* reveal the genomic elements where the fungal sRNAs come from. Sense means the sRNAs come from the same strand with the genomic elements while antisense means the opposite strand.

**Figure 2:**
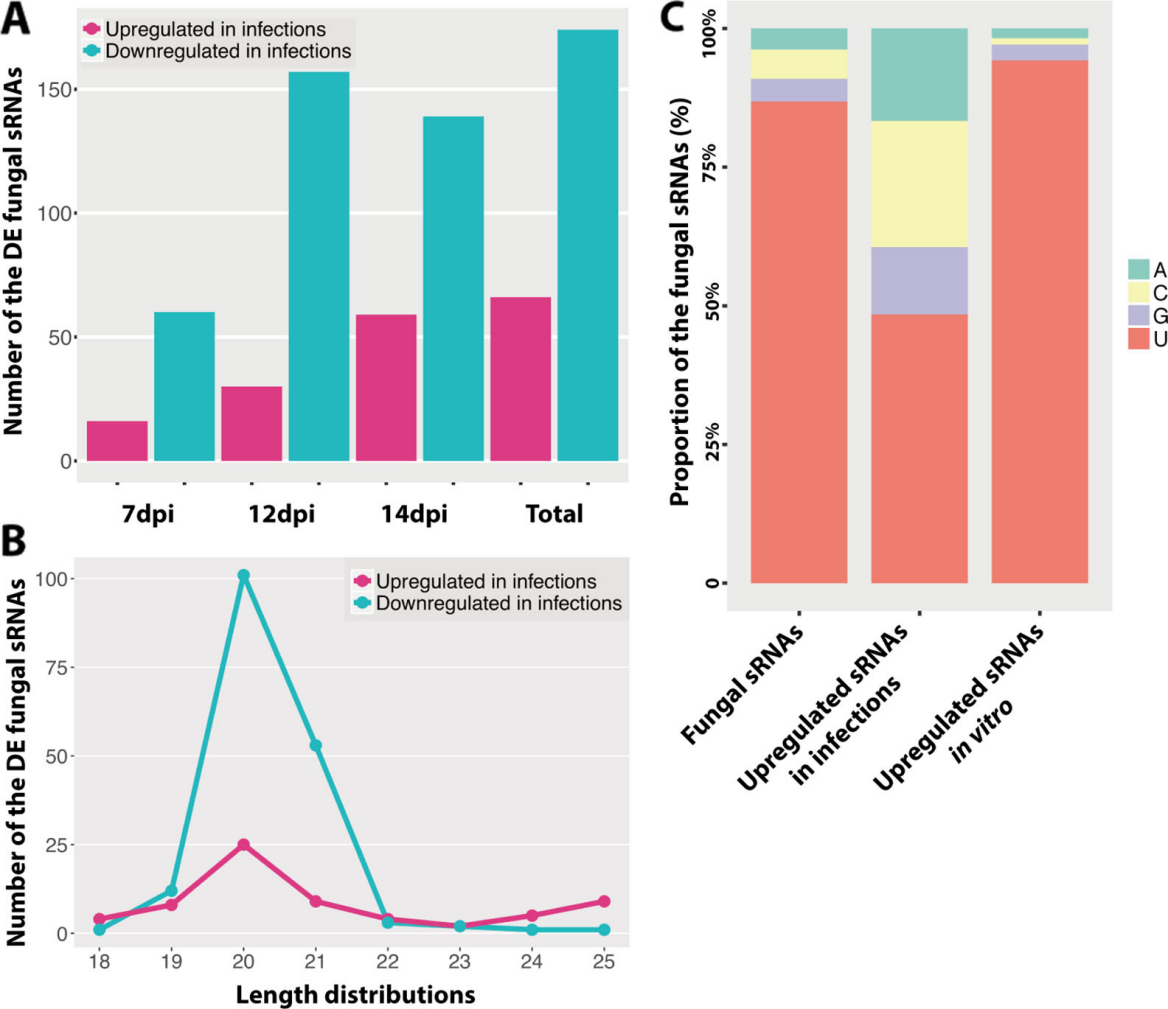
Differentially expressed fungal small RNAs (sRNAs) during the infection cycle. **(A)** Number of the differentially expressed (DE) fungal sRNAs during the infection cycle. “Total” indicates the total number of DE fungal sRNAs across all time points. **(B)** Length distributions of the DE fungal sRNAs. **(C)** Frequency of nucleotides at 5′ end of the fungal sRNAs.

Using a customized bioinformatic analysis (see *Methods* section), we predicted 662 unique *Z. tritici* RNAs in total (**Table S1**) (Shahid and Axtell, 2014; Johnson et al., 2016). We considered all the major RNAs predicted by ShortStack as the fungal sRNAs. The most abundant fungal sRNAs were of 20 and 21 nt in length (**Figure 1A**). The fungal sRNAs were originated across the fungal genome, including both core and accessory chromosomes, although most sRNAs came from chromosomes 1 and 3 (**Figure 1B**). Among the total number of fungal sRNAs, 160 were originated from gene exon regions, 19 were from gene intron regions, 343 were from transposable elements, and 212 were from the intergenic regions (**Figure 1C**). The size distribution and the chromosome of origin of these *Z. tritici* sRNAs are consistent with the previous study (Kettles et al., 2019). Besides, a similar proportion of nucleotides at 5′ end of the sRNAs was found in our analysis (**Figure 2A**).

### The Composition of Fungal sRNAs Changes During the Infection Cycle

We analyzed the differentially expressed fungal sRNAs between infected samples and *in vitro* samples to identify the infection-induced fungal sRNAs (**Table S2**). We detected that 66 fungal sRNAs were significantly upregulated in the infected samples compared to the *in vitro* samples (**Figure 2A**). These fungal sRNAs were considered as the infection-induced fungal sRNAs and therefore they may play important roles during plant infection. The number of induced fungal sRNAs was only 16 at 7 dpi, when the fungal pathogen was in the latent period and no symptoms were detected on the leaves. But this number increased to 30 at 12 dpi, when the first chlorotic symptoms appeared on the leaves, and 59 at 14 dpi, when necrotic lesions are clearly observed on the leaves (Palma-Guerrero et al., 2017). Besides, there were 174 fungal sRNAs significantly downregulated in the infection samples (**Figure 2A**).

The length and the nucleotide at 5′ end of the sRNAs are considered to determine to which AGO proteins are they binding, as it has been shown for *Arabidopsis*, and subsequently the sRNA final function (Mi et al., 2008). In our analysis, the length distributions of the differentially expressed fungal sRNAs between *in vitro* and infected samples were similar, being 20/21 nt (**Figures 1A** and **2B**). At the same time, most of the fungal sRNAs shared uracil (U) at 5′ end, which have been shown to bind preferentially to AGO1 in *Arabidopsis* (**Figure 2C**). Interestingly, the proportion of U at 5′ end changed dramatically, from 87% among all the sRNAs to 48% among the infection-induced sRNAs. Correspondingly, 23% of the infection-induced fungal sRNAs started with C, binding preferentially to AGO5 in *Arabidopsis*, and 16.7% started with A, binding preferentially to AGO2 and AGO3 in *Arabidopsis* (**Figure 2C**). This change indicates that there is different fungal RNA accumulation between growth *in vitro* and *in planta* and that the sRNAs induced during infection could have different functions from the sRNAs produced during *in vitro* growth, being part of different AGOs/sRNA complexes.

### *Z. tritici* sRNAs Were Not Detected to Cleave Wheat Transcripts

The cross-kingdom RNAi between plants and fungal pathogens has been shown in different pathosystems (Zhang et al., 2011; Weiberg et al., 2013; Wang et al., 2016; Zhang et al., 2016; Wang et al., 2017). To investigate the presence of cross-kingdom RNAi in the wheat–*Z. tritici* pathosystem, we used the predicted fungal sRNAs to detect target genes in the wheat transcriptome. We found that 33 fungal sRNAs were either highly expressed (above 100 RPM) or upregulated at 7 or 12 dpi, which were predicted to target wheat genes. Among these targeted wheat genes, 139 were significantly downregulated during the infection (**Table S3**).

**Figure 3:**
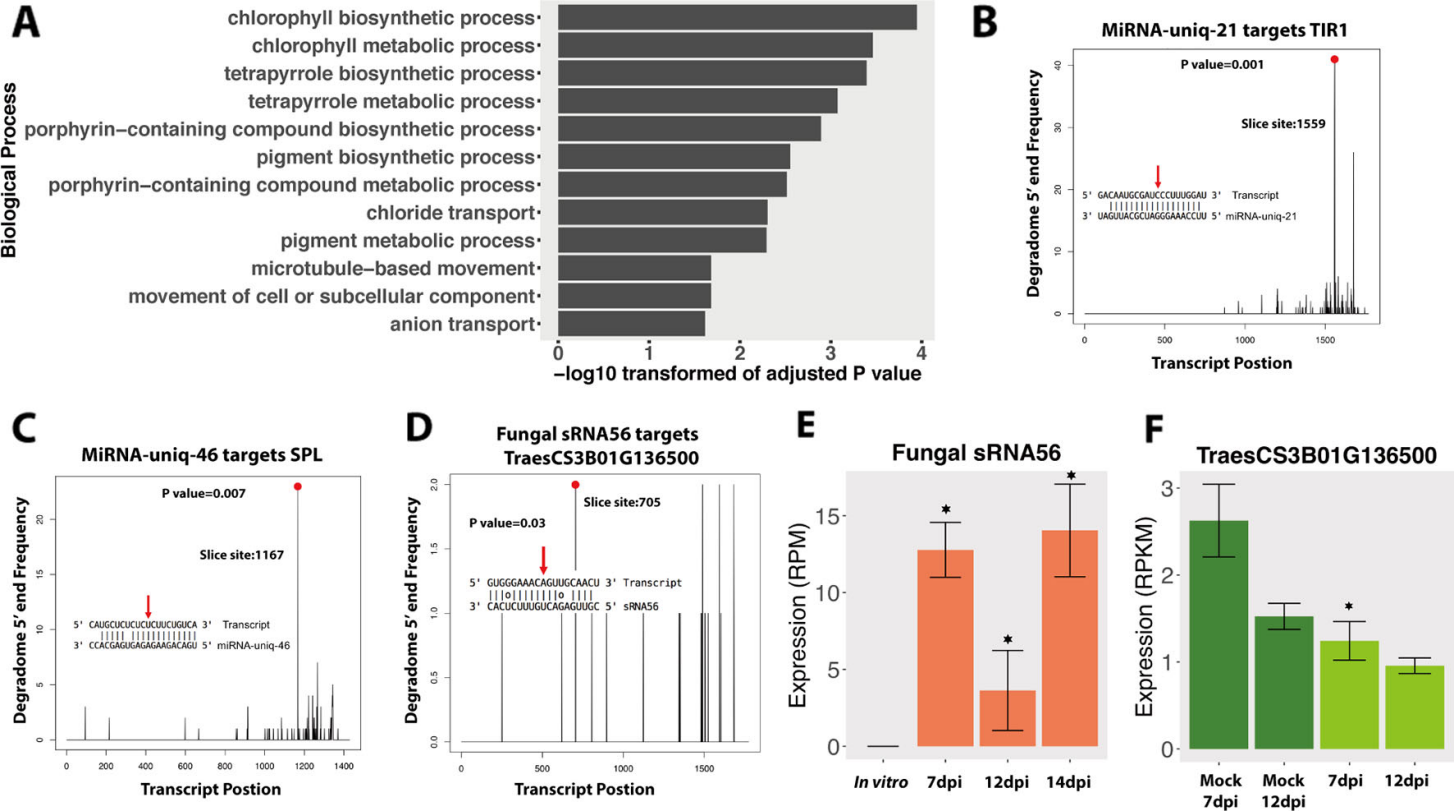
Degradome analysis of the fungal small RNAs (sRNAs). **(A)** GO enrichment of biological process of wheat genes that were downregulated and predicted to be targeted by fungal-induced sRNAs during the infections. **(B**, **C)** Positive controls of degradome sequencing results. The *x*-axis indicates the position of the target transcript and the *y*-axis indicates the degraded frequency of the target transcript. The *red point* and *red arrow* indicate the slice site from the transcript and the sRNA. The *P* value indicates the significance of the target. **(D)** Degradome results of fungal sRNA56 targeting plant gene. **(E**, **F)** Expressions of fungal sRNA56 and its target gene in plant. *Asterisk* indicates that the sRNA or the target genes are differentially expressed compared to the *in vitro* or the mock conditions, respectively.

These 139 wheat genes were significantly enriched in chlorophyll-related processes and pigment biosynthesis (**Figure 3A**), which would affect photosynthesis, the leaf color, and the plant defense ability (Menzies et al., 2016). This suggests that fungal sRNAs may regulate plant chlorophyll-related genes to reduce the plant defense ability. Interestingly, these targeted wheat genes were enriched in “microtubule-based movement” biological process, which could affect the plant intracellular transport and secretion, and therefore affect the plant response to the pathogen infection (Lee et al., 2012). Besides these, fungal sRNAs were also predicted to target wheat receptor-like kinase (RLK) genes and resistance genes, which also suggests that fungal sRNAs were trying to disturb the plant defense system (**Table S3**).

Next, we performed a degradome analysis to validate the cleavage of the candidate targeted genes. Three conditions were used in this analysis: *Z. tritici* grown *in vitro*, wheat leaves infected by *Z. tritici* (7 and 12 dpi), and uninfected mock leaves at the same time points. To determine the quality of the degradome sequencing, we first analyzed several plant miRNAs that are already known to target plant genes. In our analysis, these plant miRNAs were predicted to target and cleave plant transcripts, as previously reported, which confirmed the good quality of the degradome sequencing. For example, miRNA-uniq-21 (known as miR393) and miRNA-uniq-46 (known as miR156) were correctly found to cleave TIR1 genes and Squamosa promoter-binding-like (SPL) transcription factor genes, respectively, in our data (**Figures 3B, C**), as has been previously reported (Addo-Quaye et al., 2009).

In the degradome analysis, only one wheat gene, TraesCS3B01G136500, was found to be cleaved and downregulated by fungal sRNA56 at 7 dpi (**Figure 3D**). This wheat gene encodes a “Remodeling and spacing factor 1 (RSF-1)” protein, which is known to be involved in DNA double-strand break repair in mammals (Pessina and Lowndes, 2014). SRNA56 was induced during infection, but being low expressed (**Figure 3E**). Additionally, the significantly reduced target, TraesCS3B01G136500, was constantly very low expressed (below 3 RPKM) and the degradome coverage of the cleaved transcripts was also low, which raises doubt about it being a real target (**Figures 3D, F**). These degradome sequencing results support the recent findings from Kettles et al. showing that the IPO323 strain of *Z. tritici* could not cleave wheat transcripts during infection (Kettles et al., 2019).

**Figure 4:**
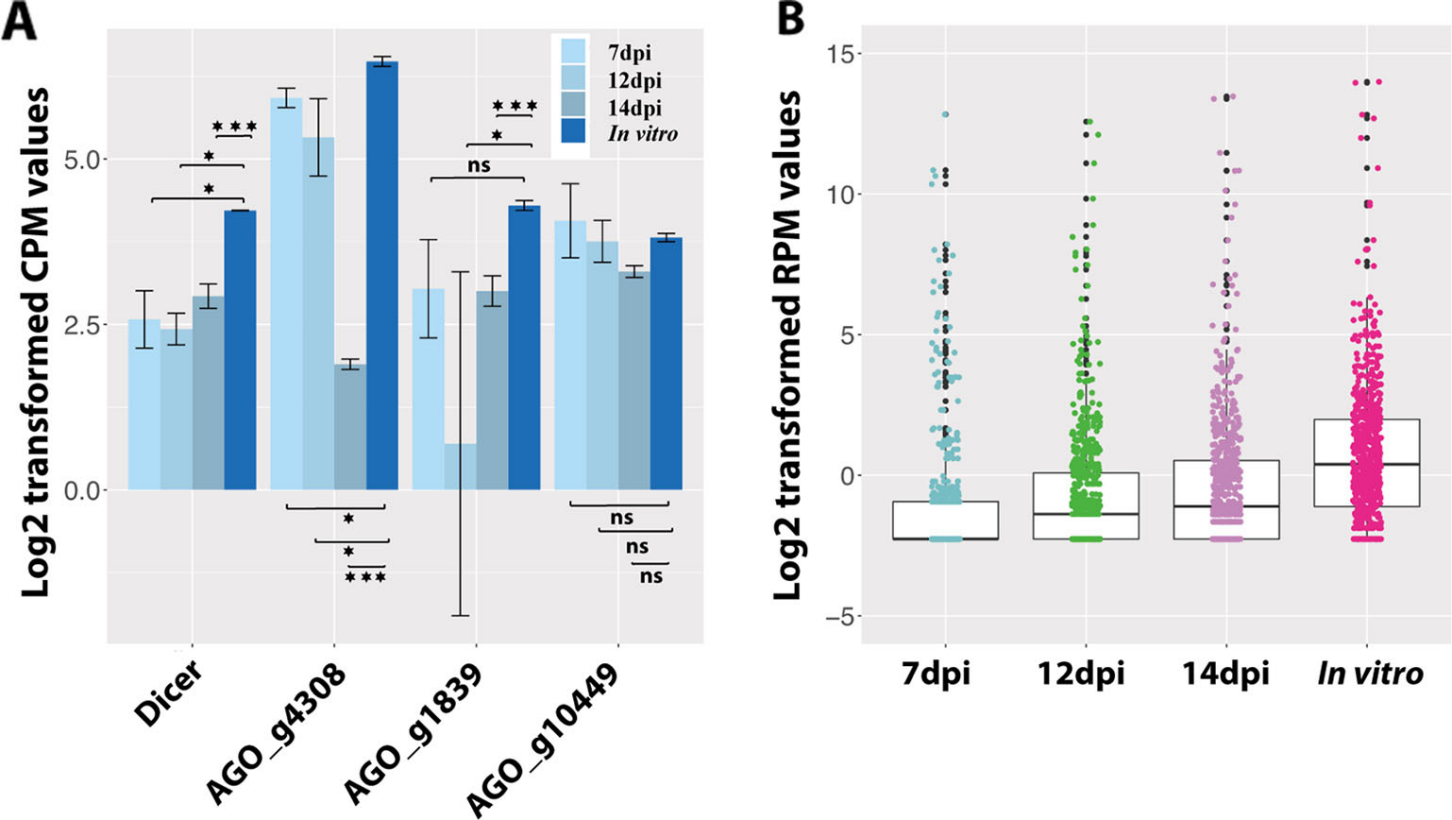
Fungal small RNAs (sRNAs) are more abundant *in vitro* than *in planta*. **(A)** Expression of the fungal Dicer gene and three AGO genes. *One asterisk* indicates that the adjusted *P* value is under 0.05 and *three asterisks* indicate that the *P* value is under 0.001. *ns* indicates no significant difference. **(B)** Average expression of all the fungal sRNAs *in planta* and *in vitro*.

We also investigated the RNAi in *Z. tritici* during the disease cycle. In total, 39 fungal sRNAs were predicted to target and downregulate the fungal own genes during the infection (**Table S4**). But none of these genes were detected to be cleaved in the degradome sequencing. Finally, we analyzed the expressions of genes involved in RNAi in *Z. tritici*. There is only one Dicer gene in the *Z. tritici* genome (g9428 from the 3D7 genome), which is consistent across five different sequenced *Z. tritici* genomes (Goodwin et al., 2011; Plissonneau et al., 2016; Plissonneau et al., 2018). Surprisingly, this Dicer gene was significantly downregulated *in planta* compared with *in vitro* (**Figure 4A**). Additionally, the sRNAs were more abundant *in vitro* than *in planta*, which could be caused by the low expression of the fungal Dicer gene (**Figure 4B**). There are four AGO genes in the *Z. tritici* genome (g4308, g1839, g10449, and g2094 of the 3D7 genome), among which the AGO gene g2094 was not expressed in any condition tested. We as well did not find any significant upregulation of AGO genes in *Z. tritici* during infection (**Figure 4A**). The downregulation pattern of RNAi-related genes in *Z. tritici* also indicates their unimportant roles during wheat infection.

### Wheat Regulates sRNAs in Response to Fungal Infections

**Figure 5:**
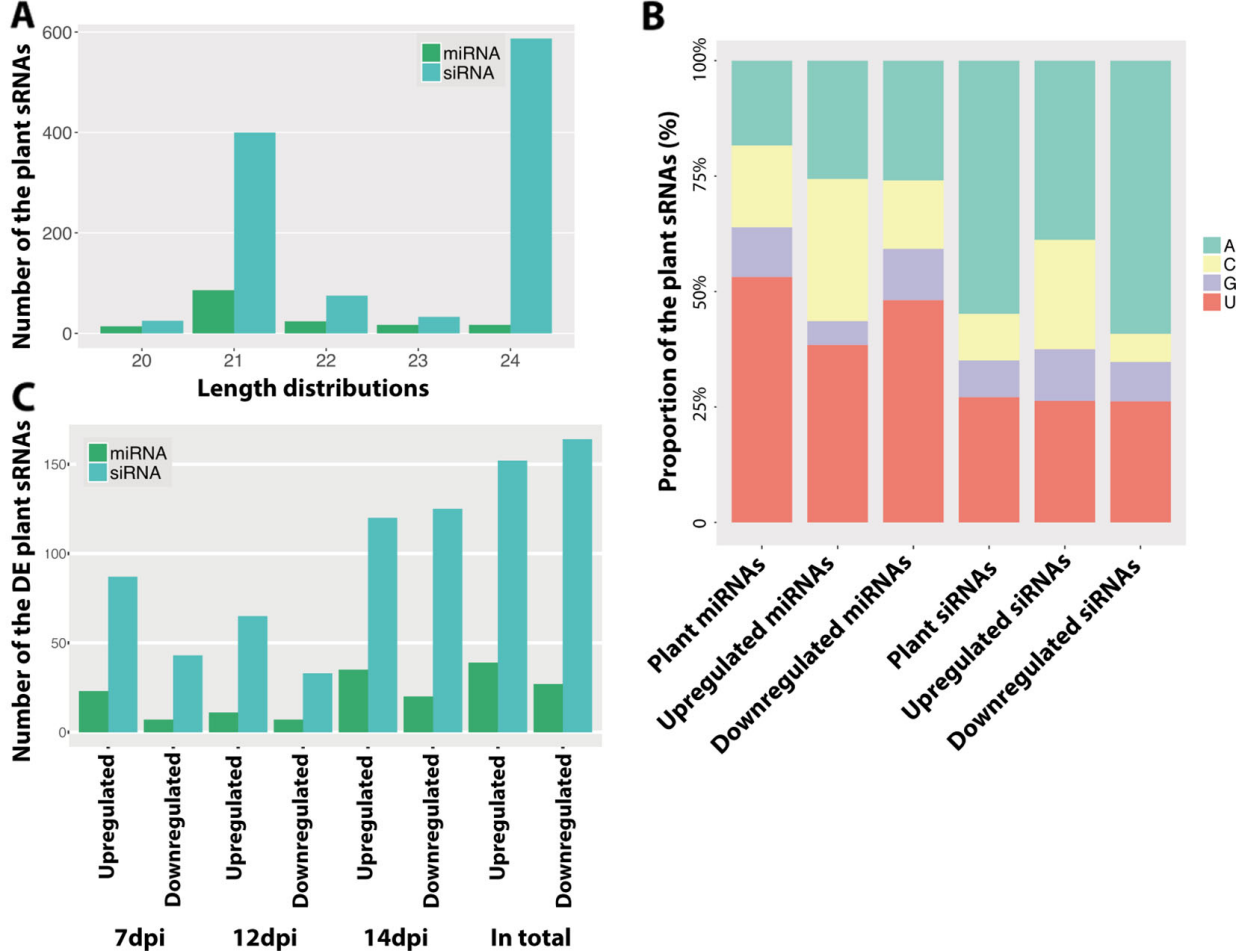
Predicted plant small RNAs (sRNAs). **(A)** Length distribution of the predicted plant sRNAs. **(B)** Frequency of nucleotides at 5′ end of plant sRNAs. *Different colors* indicate different nucleotides. **(C)** Number of the differentially expressed sRNAs between infections and mocks. *Upregulated* indicates the sRNAs are upregulated in the infections and *Downregulated* indicates the sRNAs are downregulated in the infections.

We used the same dataset to identify the wheat sRNAs and to study the roles of wheat sRNAs in the disease cycle. In total, we identified 158 wheat miRNAs and 1,120 wheat siRNA loci (**Table S5**). By blasting these predicted miRNAs against the known wheat miRNAs, we found 69 miRNAs that were already annotated in the wheat genome. The other 89 miRNAs were considered as novel wheat miRNAs (**Table S5**). Most wheat-predicted miRNAs were 21 nt in length, consistent with previous studies (**Figure 5A**) (Rajagopalan et al., 2006; Axtell, 2013). The majority of these plant miRNAs start with U at 5′ end (**Figure 5B**), which should favor binding the plant AGO1 proteins (Mi et al., 2008). Unlike the miRNAs, most of the wheat siRNAs were 24 nt in length and started with A (**Figures 5A**, **B**). These 24-nt siRNAs have been shown to act by RdDM in *Arabidopsis* (Matzke and Mosher, 2014), being preferentially loaded in AGO4 (Mi et al., 2008).

Plants can regulate the sRNAs, especially miRNAs, to respond to pathogens, which is thought to be a fast and efficient immune response (Fei et al., 2013). In order to determine if the infection induces variations in sRNA accumulation, we compared the expression of all the plant sRNAs between infected and mock samples at every time point. Like for the fungus, the infection-induced plant sRNAs also exhibited different frequencies of nucleotides at 5′ end (**Figure 5B**), which suggests a different sRNA production during infections.

**Figure 6:**
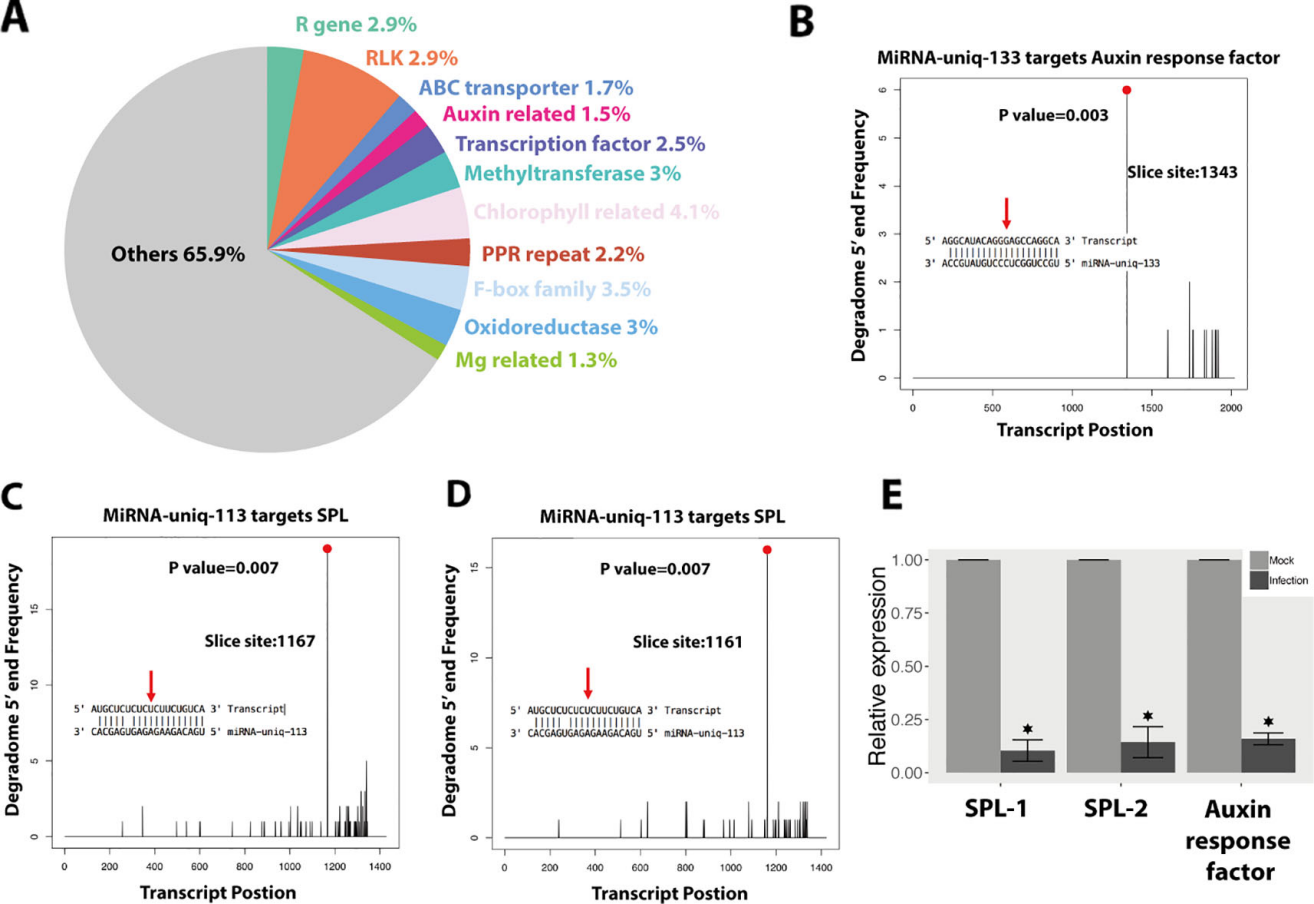
Wheat induces small RNAs (sRNAs) to regulate wheat genes as an immune response against *Z. tritici*. **(A)** Functions of the wheat genes, which were downregulated and predicted to be targeted by wheat-induced sRNAs. **(B**–**D)** Degradome analysis of the wheat genes targeted by miRNA-uniq-133 and miRNA-uniq-113. **(E)** Relative expression of miRNA-133 and miRNA-113 targeted genes in infections compared to mocks. Each value represents the average of three biological replicates, with three technical replicates per sample. *Asterisk* indicates significant downregulation during fungal infection (*P* value under 0.05).

We found that 191 plant sRNAs were significantly upregulated in the infections, among which 39 were miRNAs (15 novel miRNAs) and 152 were siRNAs (**Figure 5C** and **Table S6**). Among these induced or extremely highly expressed (RPM above 100) plant sRNAs, 176 sRNAs (47 miRNAs and 129 siRNAs) were predicted to target and silence 690 wheat genes (**Table S7**). Interestingly, 20 (3%) of these candidates targeted genes were resistance genes and 77 (11.2%) were RLKs, which are considered to play central roles in plant immunity. Besides, 12 (2%) genes encode ABC transporters and 11 (1.5%) genes encode auxin response factor, which are also genes involved in the plant immune system (**Figure 6A**).

**Figure 7:**
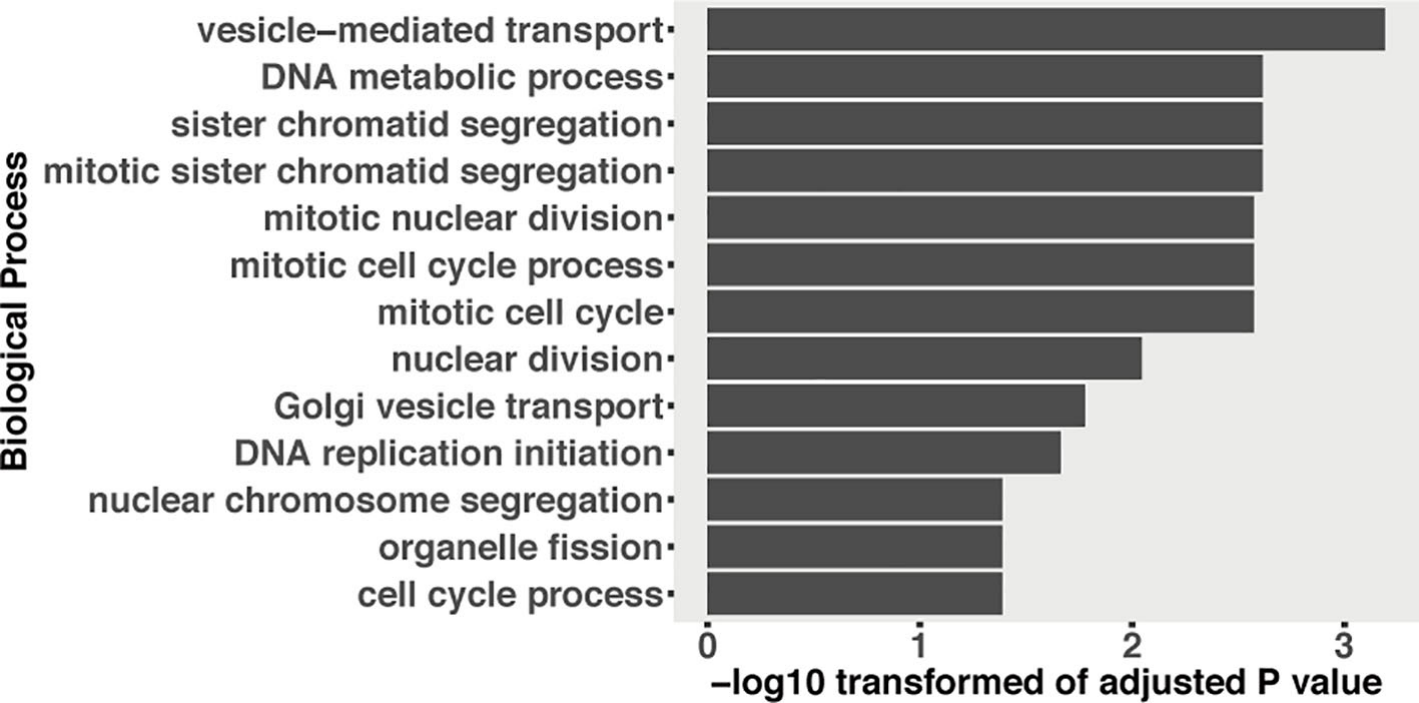
GO enrichment of fungal genes, which were downregulated and predicted to be targeted by induced plant sRNAs during the infection.

We confirmed the cleavage of three transcripts among these 690 genes (**Figures 6B–D**). MiRNA-uniq-133 (miRNA160 family) was detected to silence TraesCS6A01G222300.1, which encodes an auxin response factor (**Figures 6B** and **S1**). Two SPL genes were negatively regulated by miRNA-uniq-113 (belonging to miRNA156 family), which was upregulated at 7 and 12 dpi (**Figures 6C**, **D** and **S1**). The expressions of these two miRNAs were confirmed by Northern blot analysis (**Figure S2**). The downregulation of these target genes during the fungal infection was confirmed both by RNA-seq data and qPCR experiments (**Figures 6E** and **S1**).

In plant–pathogen interactions, plants can as well export miRNAs into pathogen cells to influence pathogen virulence (Wang et al., 2016). We predicted 190 wheat sRNAs that were upregulated during the infections or extremely highly expressed (RPM above 100) and could target and downregulate 1,115 fungal genes (**Table S8**). Interestingly, these targeted fungal genes were significantly enriched in “vesicle-mediated transport” GO term (**Figure 7**). We detected that two of these 1,115 fungal genes could be cleaved by wheat sRNAs (**Figure S3**). One gene is g1915, which encodes an antibiotic biosynthesis monooxygenase domain (ABM) protein. The other gene is g9791, which encodes FAD-dependent oxidoreductase. These two fungal genes were downregulated at 12 dpi (only significant for g1915) and recovered their expressions from 14 dpi (**Figure S3**). However, it has to be noted that the degradome coverage over those genes was very low and the slice site of the g1915 transcript belongs to category 3 (**Figure S3**), which suggests that it is not a real target.

## Discussion

In this study, we combined sRNA-seq, mRNA-seq, and degradome sequencing from samples covering key stages during the infection cycle to analyze the role of sRNAs in the wheat and *Z. tritici* pathosystem. We found that both *Z. tritici* and wheat sRNAs do not play important roles in a cross-kingdom manner, as shown by degradome sequencing, and supports the recently published findings for the *Z. tritici* sRNAs (Kettles et al., 2019). These two independent studies used two different *Z. tritici* strains (IPO323 and 3D7) and two different wheat cultivars (Bobwhite and Drifter) and got the same findings, indicating that cross-kingdom RNAi may not play important roles in the wheat and *Z. tritici* interaction. Additionally, we also analyzed wheat sRNAs during the disease cycle and we show that wheat can regulate wheat sRNAs in response to *Z. tritici* infections.

### RNAi Does not Play Important Roles in *Z. tritici* During Wheat Infection

In this work, we predicted *Z. tritici* sRNAs during infection cycle. Interestingly, even at 7 dpi, when the fungus was in latent period and no symptoms are detected on wheat leaves, we still detected fungal sRNAs. The concentrations and expressions of *Z. tritici* sRNAs were lower at 7 dpi compared with the other time points, probably due to the low fungal biomass at this stage. Despite this, we found induced *Z. tritici* sRNAs at 7 dpi too. Fungal sRNAs can hijack the plant host RNAi machinery to silence plant defense-related genes and facilitate fungal infection (Weiberg et al., 2013; Wang et al., 2017). However, we could not find any cleavage of wheat transcripts caused by the fungal sRNAs in the degradome sequencing. We only detected one wheat gene, encoding the RSF-1 protein, in the degradome data, which was predicted to be cleaved by fungal sRNA56. But the low expressions of sRNA56 and the low frequency of the cleaved transcript from the target gene suggested it was not a real target, at least in the canonical way (**Figures 3D–F**).

We also predicted 39 fungal sRNA that could regulate fungal genes *in planta*. But none of these targeted fungal genes were detected to be cleaved in the degradome sequencing. The fungal Dicer gene was barely expressed in the infections, which led to a decrease of sRNA genesis by the fungus (**Figure 4**). This is supported by the lower expressions of sRNAs observed *in planta* than *in vitro*. In addition, deletion of the *Z. tritici* Dicer gene has been found to have no effect on fungal virulence (Kettles et al., 2019). These results, together with our findings, suggest minor roles of the *Z. tritici* Dicer for fungal virulence. These data suggest that fungal sRNAs are not cleaving gene transcripts during the wheat–*Z. tritici* interaction.

These results are consistent with the recent study by Kettles et al. (2019), in which they show that *Z. tritici* sRNAs could not silence wheat genes in a cross-kingdom manner during infection. In these two independent studies, two different *Z. tritici* strains have been used to infect two different wheat cultivars, obtaining the same results. Additionally, our degradome sequencing data support the findings validated by molecular studies in the previous study (Kettles et al., 2019), confirming the unimportant roles of *Z. tritici* sRNAs in cross-kingdom RNAi.

However, even if the *Z. tritici* sRNAs do not play a direct role in the interaction with wheat, they may play a role at specific developmental stages of the fungus, as has been shown for other plant pathogenic fungi (Kim et al., 2015; Raman et al., 2017; Zeng et al., 2018). Also, we cannot discard the possibility that *Z. tritici* sRNAs may be acting at the transcriptional or translational level.

### Wheat sRNAs are Involved in the Response to *Z. tritici* Infection

Previous studies have reported that plants can employ sRNAs as a rapid defense response against pathogens. These plant sRNAs could regulate auxin-related genes, MYB transcription factors, and as well R genes in plant (Achard et al., 2007; Chen et al., 2007; Navarro et al., 2008; Grant and Jones, 2009; Zhai et al., 2011; Shivaprasad et al., 2012; Fei et al., 2013; Samad et al., 2017).

Here, we found that 191 wheat sRNAs were induced during fungal infection, the composition of which were quite different from the plant sRNAs expressed in the mocks (**Figure 5B**). These induced wheat sRNAs were predicted to target and silence 690 wheat genes (**Table S7**), including R genes, RLKs, ABC transporters, and auxin response factors, which are known to play central roles in the plant immunity (**Figure 6A**). Besides, many transcription factor genes were also predicted to be targeted by these induced sRNAs, including SPL transcription factors, F-box domain proteins, MYB transcription factors, and basic/helix-loop-helix (bHLH) proteins (**Figure 6A**). We confirmed the cleavage of three transcripts, which encode on auxin response factor and two SPL proteins. (**Figures 6**, **S1** and **S2**). The induced sRNA-mediated auxin-related gene here is consistent with the response observed during plant infection by bacterial pathogens (Navarro et al., 2008). When plants are under biotic stress, sRNAs are induced to regulate transcription factors as an immune response (Tsuda and Somssich, 2015). Here, we confirmed that SPL transcription factors are mainly involved in this sRNA-mediated immune response. Thus, we show clear evidence that wheat will induce a group of specific sRNAs to regulate wheat genes as an immune response to *Z. tritici*.

In HIGS, plants can transport RNA modules and target pathogen genes to reduce pathogen virulence (Nowara et al., 2010; Baulcombe, 2015). It has been proven recently that plants use extracellular vesicles to send sRNAs into fungal cells (Cai et al., 2018). These finding suggest that the RNA molecules can be transported to fungal pathogens naturally. We predicted 1,115 fungal genes that could be targeted by 190 wheat sRNAs. These wheat sRNAs were either upregulated or extremely highly expressed during the infections (**Table S8**). Interestingly, the targeted fungal genes were enriched in the “vesicle-mediated transport” GO term (**Figure 7**). Fungal extracellular vesicles (EVs) are essential to transport proteins, glycan pigments, nucleic acids, and lipids (Rodrigues et al., 2015; Rodrigues and Casadevall, 2018). Fungal EVs are also responsible to pathogenesis during the infection (Rodrigues et al., 2007; Albuquerque et al., 2008; Rodrigues et al., 2008; Vallejo et al., 2011; Vargas et al., 2015), including mediating the transport of virulent effectors into plant cells (Rodrigues et al., 2008). A recent study also proved that plant miRNAs can be transported by EVs into fungal cells to affect fungal virulence (Cai et al., 2018). Here, our results suggest that wheat sRNAs may target fungal EV-related genes to interfere fungal virulence. However, we only detected two fungal genes that were cleaved by the wheat sRNAs (**Figure S3**). Fungal g1915, which encodes an ABM protein, involved in diverse biological processes, including metabolism, transcription, translation, and biosynthesis of secondary metabolites, was cleaved by plant siRNA180. The other one is fungal g9791, which encodes FAD-dependent oxidoreductase, which was targeted by plant miRNA-uniq-21. These two fungal genes play fundamental roles in fungal growth and they are downregulated at the beginning of the disease cycle. However, these two fungal genes were not completely silenced, and the expression of these two genes was recovered at 14 dpi, when the fungus started the necrotrophic growth phase. But as we discussed before, the degradome results did not provide support for the cleavage of the transcripts from these two genes. These results suggest that wheat is not able to use sRNAs to silence fungal genes. These results are supported by the finding that dsRNAs generated from RNA virus vectors *in planta* are not effective in triggering gene silencing in *Z. tritici* during the fungal infection (Kettles et al., 2019).

In conclusion, our results support the recent finding that there is no natural cross-kingdom RNAi causing the cleavage of gene transcripts between these two interacting species. However, we found that wheat can use wheat sRNAs to regulate the plant defenses during *Z. tritici* infection. These findings contribute to improve our understanding of the hidden interactions in this pathosystem.

## Data Availability Statement

All the raw sequencing data were deposited into the NCBI Short Read Archive under the accession number SRP154808.

## Author Contributions

XM, JW, and JP-G conceived and designed experiments. XM was responsible for the bioinformatics analysis. JW, XM, and JP-G were responsible for the experimental validations. XM, and JP-G wrote the paper.

## Funding

XM is funded by a PSC-Syngenta PhD fellowship. Laboratory facilities were provided by the Genetic Diversity Center (GDC) of ETH Zurich.

## Conflict of Interest

The authors declare that the research was conducted in the absence of any commercial or financial relationships that could be construed as a potential conflict of interest.
