# Supplementary figures and images for: Small RNA Bidirectional Crosstalk During the Interaction Between Wheat and Zymoseptoria tritici
Source: Front Plant Sci. 2020 Jan 8;10:1669. doi: 10.3389/fpls.2019.01669 (PMC6960233; doi:10.3389/fpls.2019.01669)

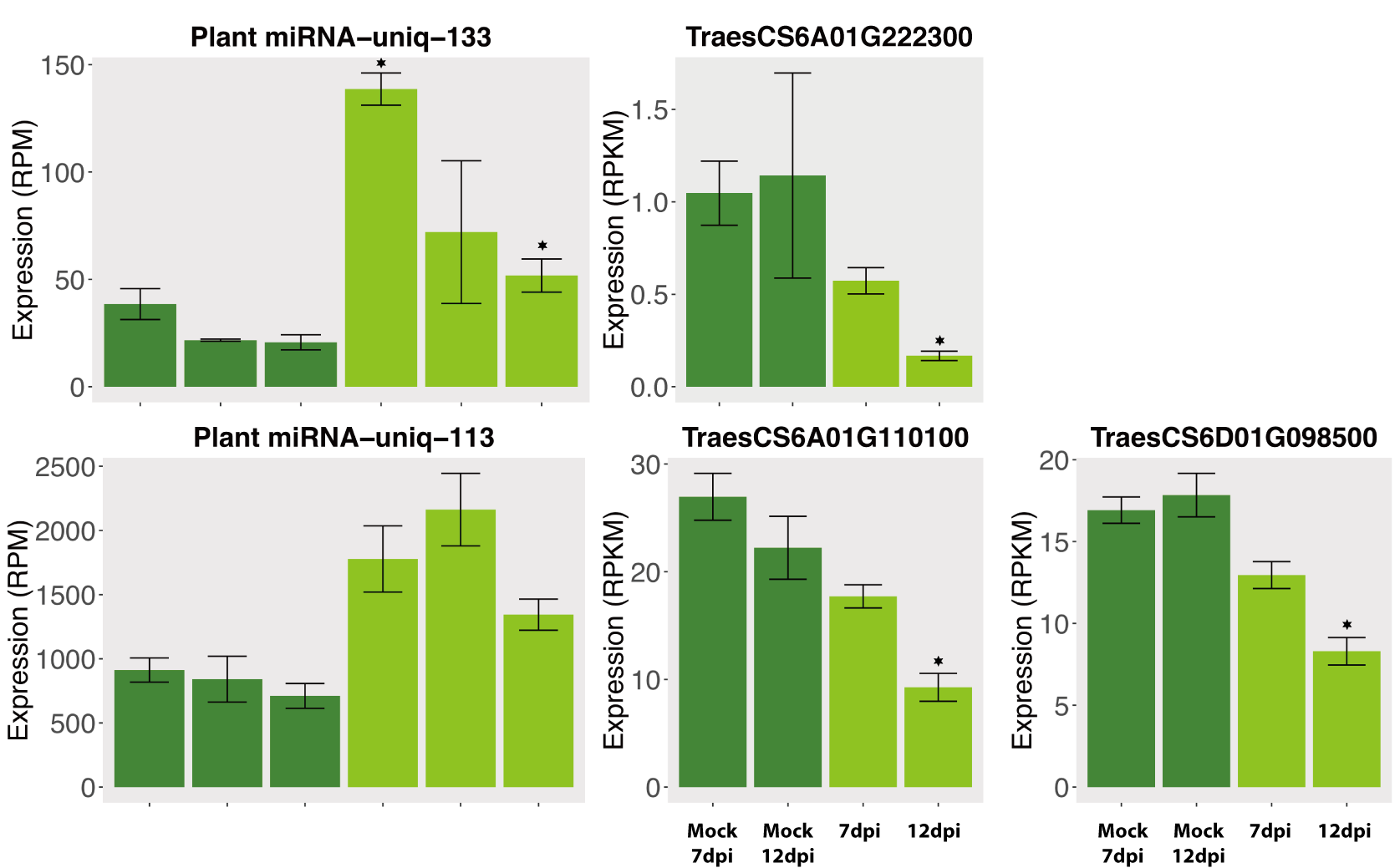

Supplement: Supplementary file 9 [file Image_1.tif]

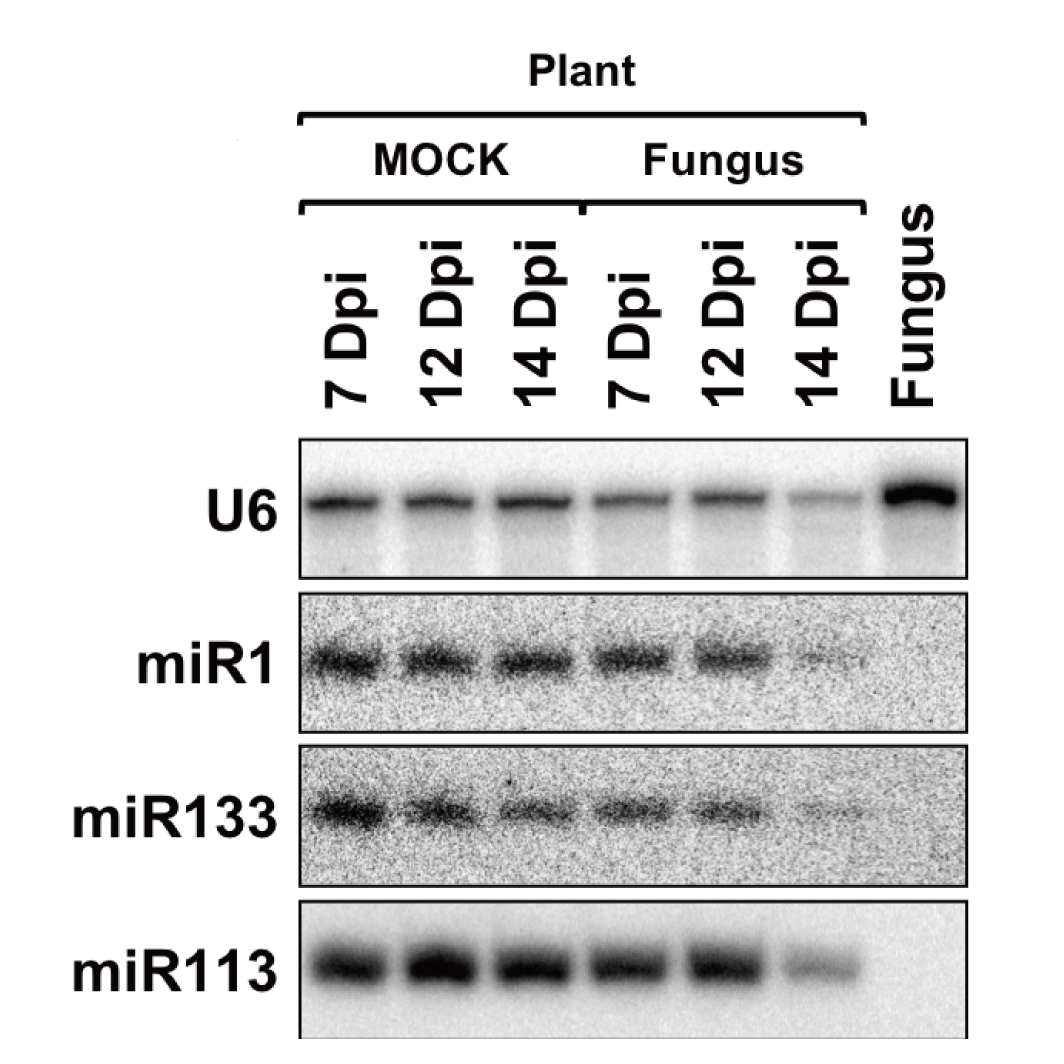

Supplement: Supplementary file 10 [file Image_2.tif]

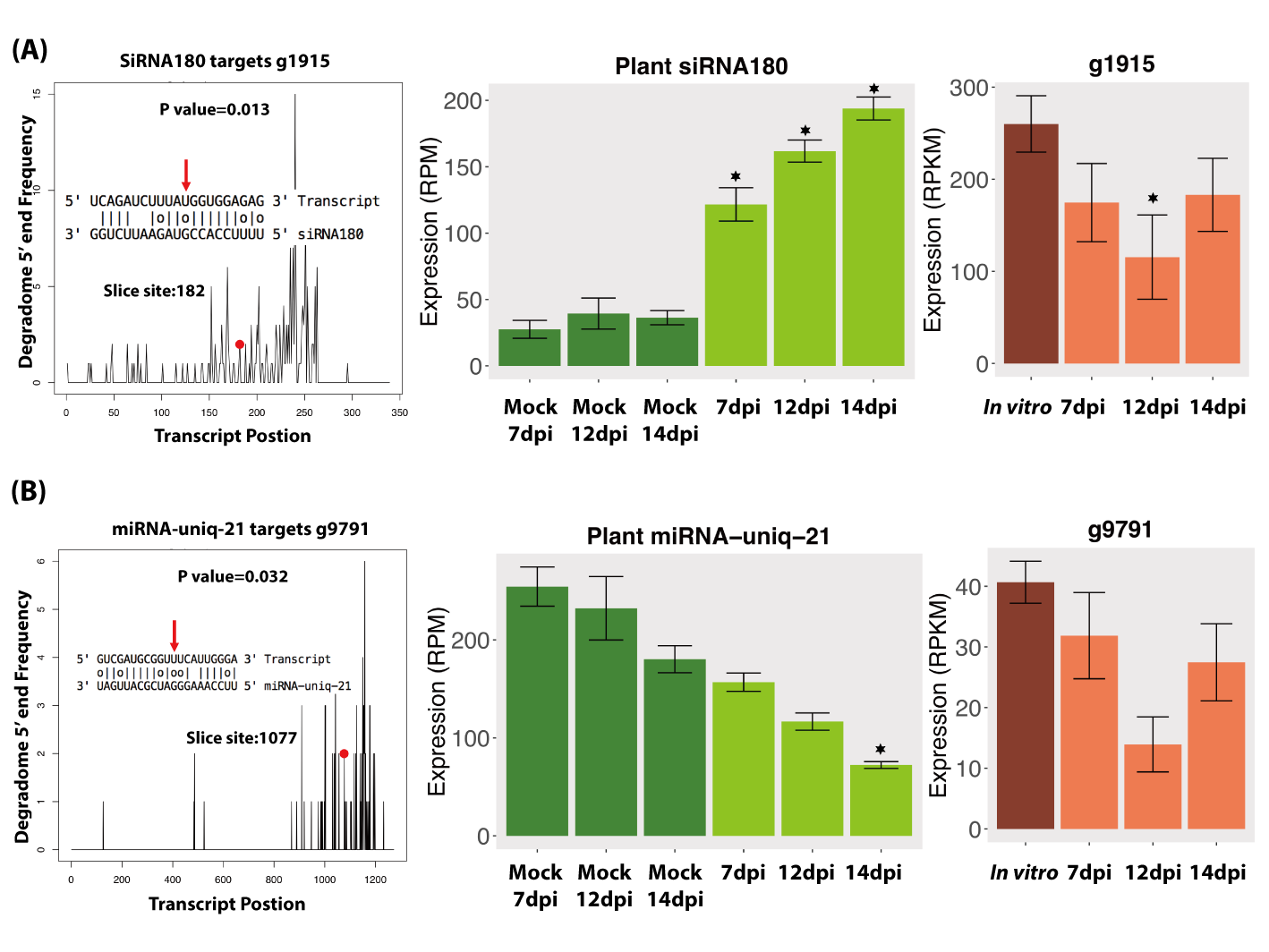

Supplement: Supplementary file 11 [file Image_3.tif]
